# Supplementary material for: Improved Quantitative Parameter Estimation for Prostate T2 Relaxometry using Convolutional Neural Networks
Source: medRxiv. 2023 Mar 29:2023.01.11.23284194. Originally published 2023 Jan 12. Preprint. [Version 2] doi: 10.1101/2023.01.11.23284194 (PMC9882442; doi:10.1101/2023.01.11.23284194)
Supplement: 1 [file NIHPP2023.01.11.23284194v2-supplement-1.pdf]

medRxiv preprint doi: https://doi.org/10.1101/2023.01.11.23284194; this version posted March 29, 2023. The copyright holder for this preprint (which was not certified by peer review) is the author/funder, who has granted medRxiv a license to display the preprint in perpetuity.
It is made available under a CC-BY 4.0 International license .

Supplemental Data

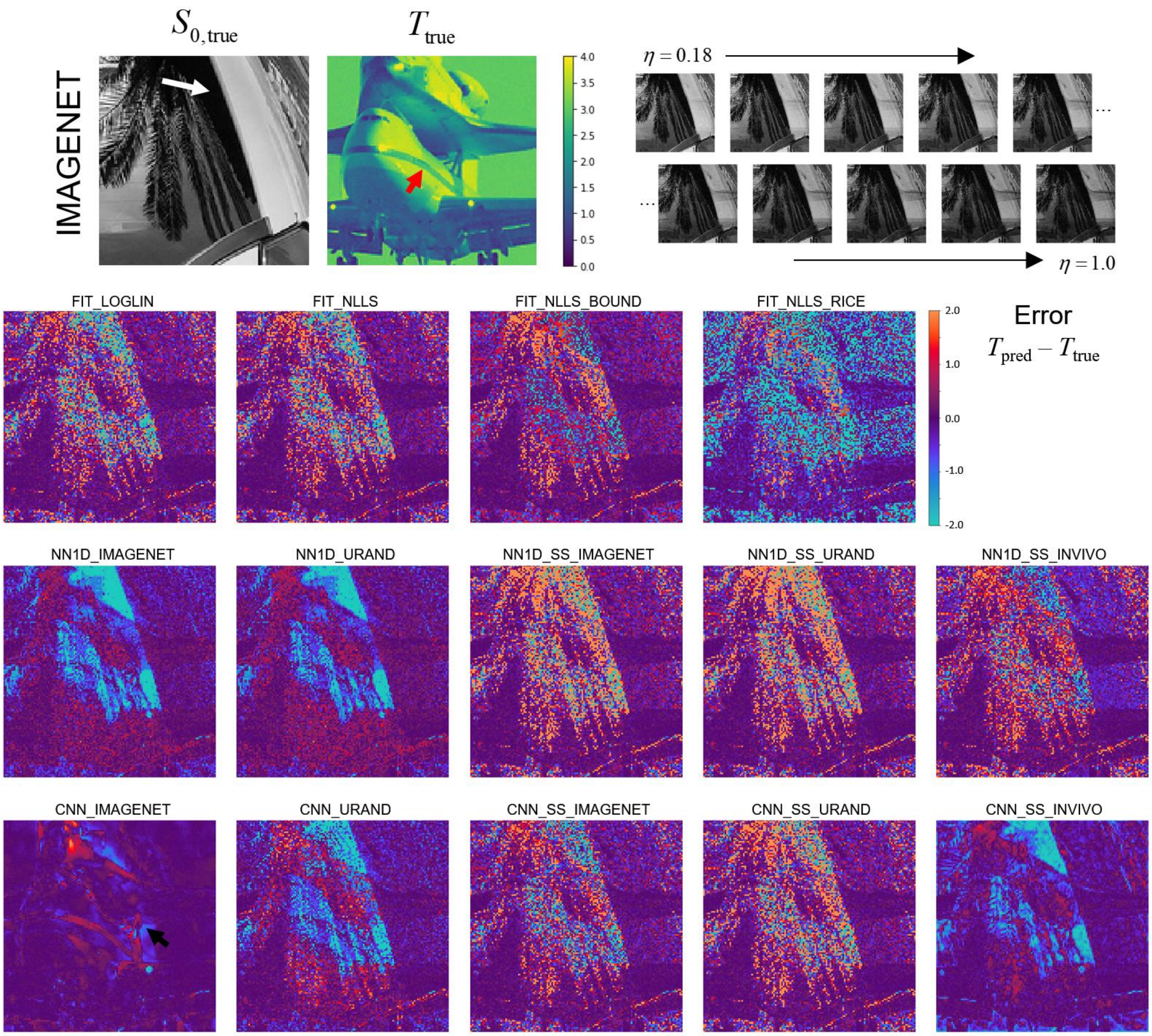

Figure S1 - Image maps showing the signed error between predicted and true T maps for each of the methods, using the example case provided in figure 3. Red pixels indicate overestimation of T (positive errors) while blue pixels indicate underestimation. All values in normalized T units.

medRxiv preprint doi: https://doi.org/10.1101/2023.01.11.23284194; this version posted March 29, 2023. The copyright holder for this preprint (which was not certified by peer review) is the author/funder, who has granted medRxiv a license to display the preprint in perpetuity.
It is made available under a CC-BY 4.0 International license .

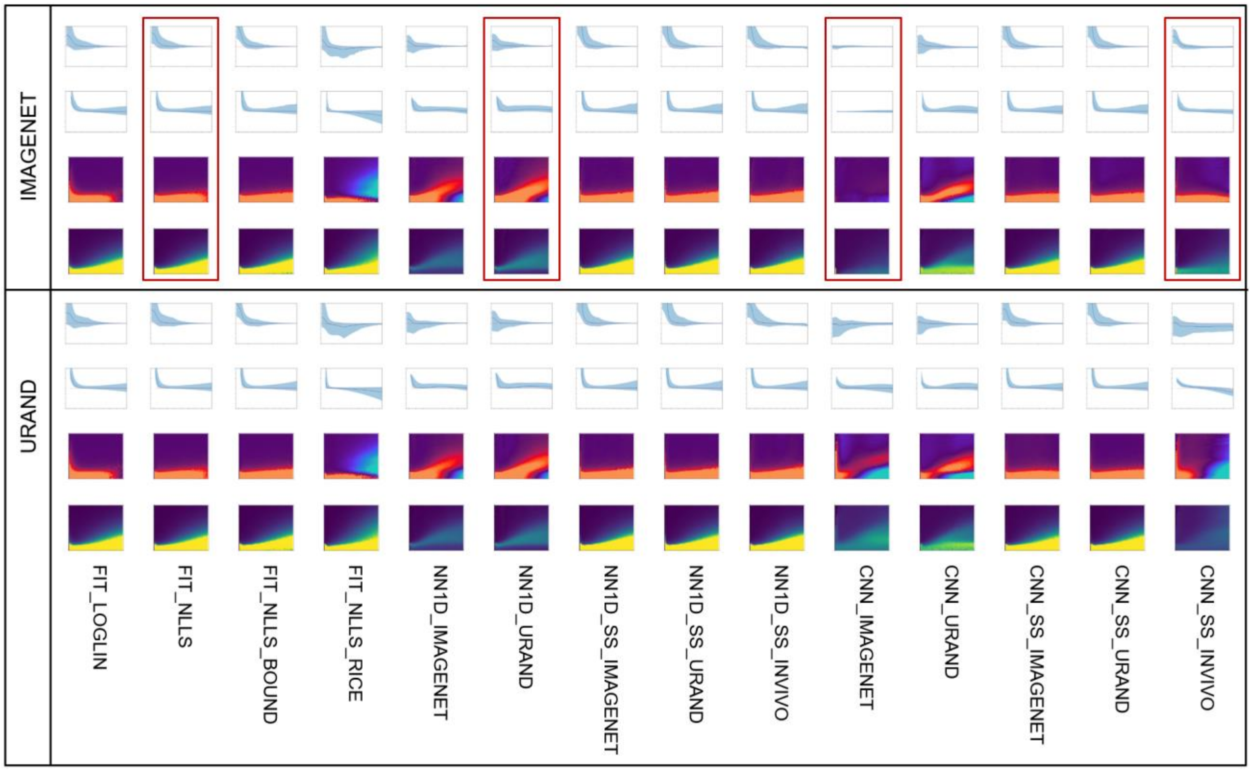

Figure S2: Expansion of figure 5, showing T-estimation error (T_{err}=T_{pred}-T_{true}) with all 14 methods on both synthetic datasets. The four cases shown in Figure 4 are outlined in red. An ideal estimator of T would be deep blue (uniformly zero error) in the colormaps, independent of both SNR and T_{true}; CNN_IMAGENET best approximates this ideal method when evaluated on the IMAGENET test dataset. See Figure 4 for further explanation and axis labeling.

medRxiv preprint doi: https://doi.org/10.1101/2023.01.11.23284194; this version posted March 29, 2023. The copyright holder for this preprint (which was not certified by peer review) is the author/funder, who has granted medRxiv a license to display the preprint in perpetuity.
It is made available under a CC-BY 4.0 International license .

Dataset	Method	Bias	Precision	Error	SSIM
IMAGENET_TEST_1K	FIT_LOGLIN	0.038	0.109	0.416	0.146
	FIT_NLLS	0.063	0.143	0.383	0.160
	FIT_NLLS_BOUND	0.081	0.191	0.363	0.257
	FIT_NLLS_RICE	-0.173	0.282	0.549	0.149
	NN1D_IMAGENET	0.042	0.149	0.340	0.342
	NN1D_URAND	0.083	0.182	0.358	0.330
	NN1D_SS_IMAGENET	0.094	0.224	0.394	0.159
	NN1D_SS_URAND	0.089	0.229	0.395	0.157
	NN1D_SS_INVIVO	0.082	0.233	0.397	0.197
	CNN_IMAGENET	**-0.002**	**0.017**	**0.093**	**0.678**
	CNN_URAND	**0.002**	0.157	0.336	0.303
	CNN_SS_IMAGENET	0.069	0.198	0.374	0.211
	CNN_SS_URAND	0.078	0.228	0.391	0.176
	CNN_SS_INVIVO	**-0.002**	0.127	0.248	0.415
URAND_TEST_1K	FIT_LOGLIN	0.071	0.100	0.480	0.158
	FIT_NLLS	0.079	0.130	0.457	0.149
	FIT_NLLS_BOUND	0.090	0.147	0.393	0.613
	FIT_NLLS_RICE	-0.203	0.085	0.621	0.359
	NN1D_IMAGENET	**0.001**	**0.016**	0.383	0.731
	NN1D_URAND	0.038	0.037	0.383	**0.752**
	NN1D_SS_IMAGENET	0.111	0.196	0.475	0.146
	NN1D_SS_URAND	0.105	0.196	0.480	0.124
	NN1D_SS_INVIVO	0.084	0.191	0.480	0.483
	CNN_IMAGENET	-0.089	0.055	0.434	0.647
	CNN_URAND	-0.023	0.036	**0.355**	0.711
	CNN_SS_IMAGENET	0.083	0.173	0.437	0.499
	CNN_SS_URAND	0.100	0.189	0.469	0.275
	CNN_SS_INVIVO	-0.301	0.097	0.479	0.571

Table S1 – Numerical values of the primary metrics plotted in Figure 4. The best values for each dataset (lowest bias, precision, and overall error; highest SSIM) are shown in **bold**.
