# Supplementary material for: Improved Quantitative Parameter Estimation for Prostate T2 Relaxometry using Convolutional Neural Networks
Source: medRxiv. 2023 Jan 12:2023.01.11.23284194. Preprint. [Version 1] doi: 10.1101/2023.01.11.23284194 (PMC9882442; doi:10.1101/2023.01.11.23284194)
Supplement: Supplementary file 1 [file NIHPP2023.01.11.23284194v1-supplement-1.pdf]

medRxiv preprint doi: https://doi.org/10.1101/2023.01.11.23284194; this version posted January 12, 2023. The copyright holder for this preprint (which was not certified by peer review) is the author/funder, who has granted medRxiv a license to display the preprint in perpetuity.
It is made available under a CC-BY 4.0 International license .

Supplemental Data

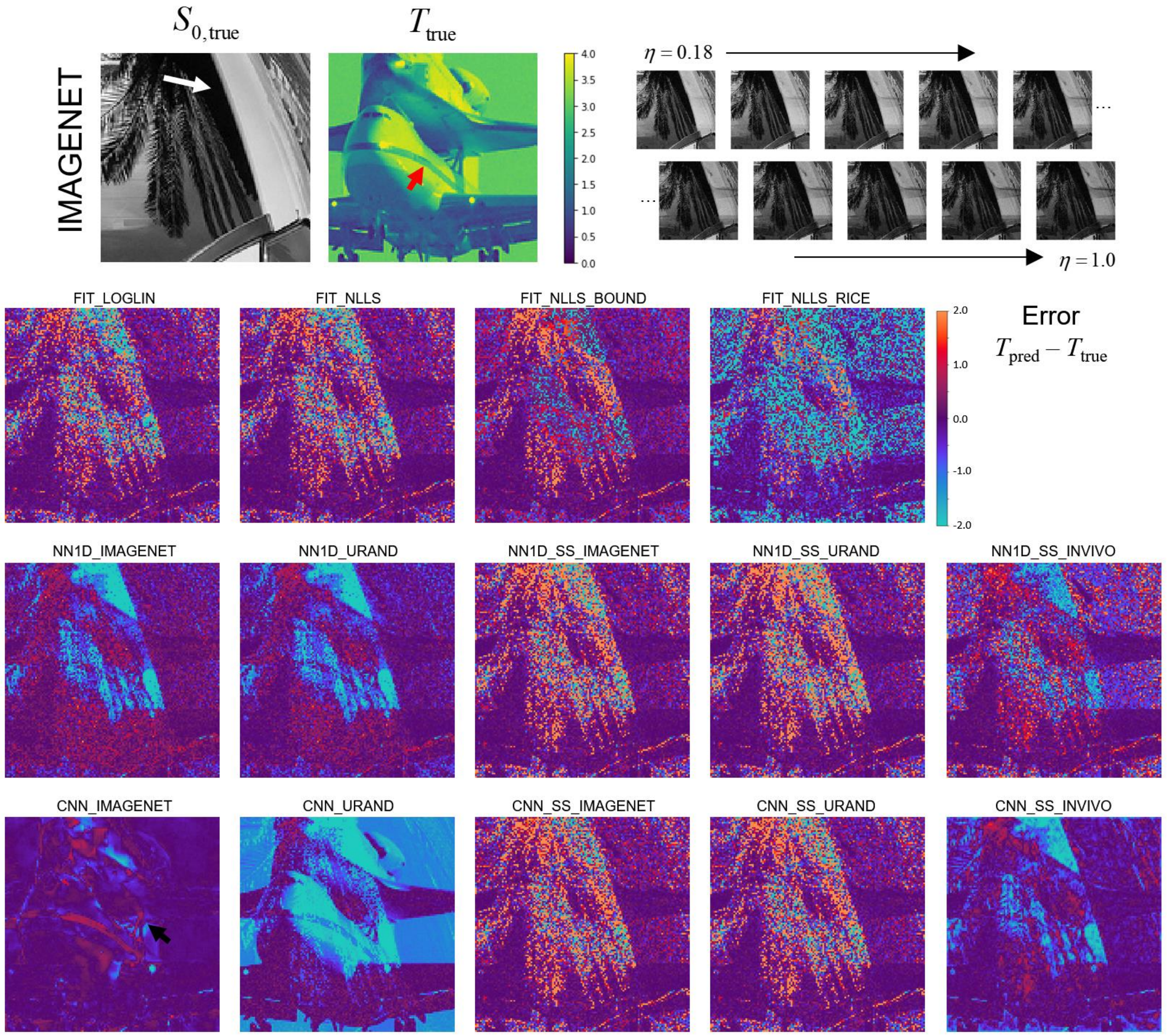

Figure S1 - Image maps showing the signed error between predicted and true T maps for each of the methods, using the example case provided in figure 3. Red pixels indicate overestimation of T (positive errors) while blue pixels indicate underestimation. All values in normalized T units.

medRxiv preprint doi: https://doi.org/10.1101/2023.01.11.23284194; this version posted January 12, 2023. The copyright holder for this preprint (which was not certified by peer review) is the author/funder, who has granted medRxiv a license to display the preprint in perpetuity.
It is made available under a CC-BY 4.0 International license .

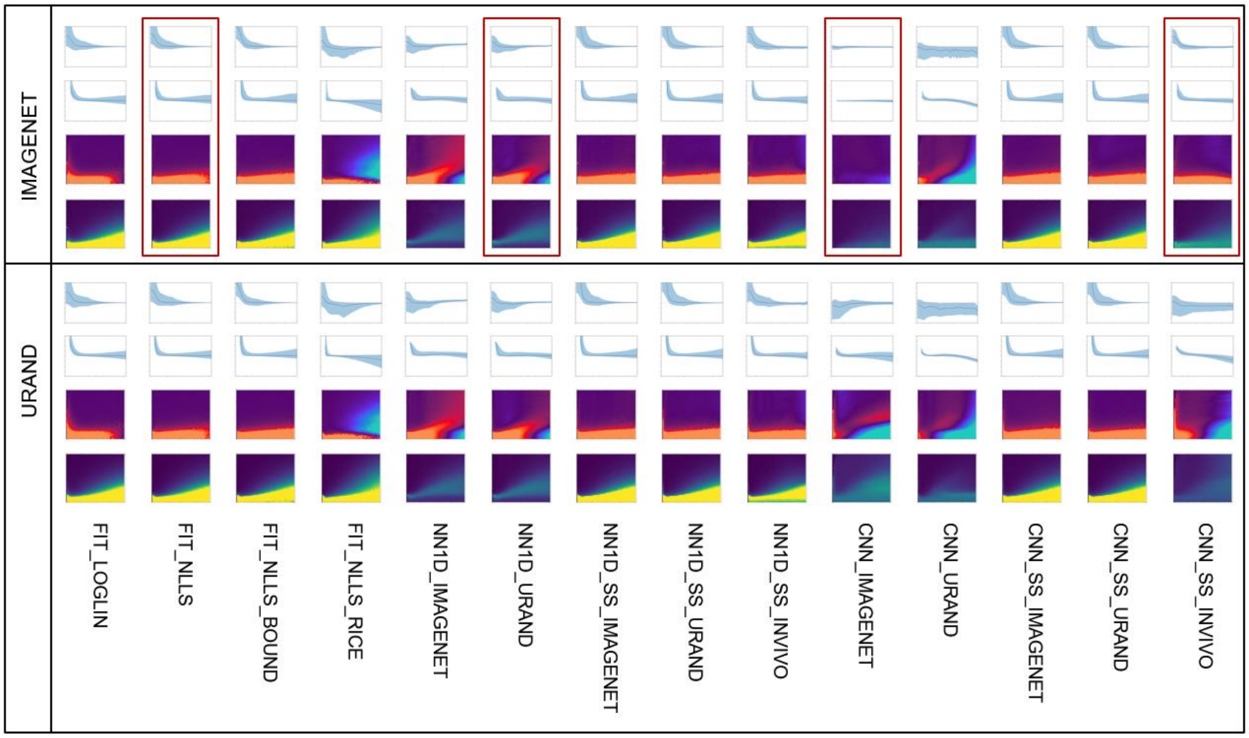

Figure S2: Expansion of figure 5, showing T-estimation error (T_{err}=T_{pred}-T_{true}) with all 14 methods on both synthetic datasets. The four cases shown in Figure 4 are outlined in red. An ideal estimator of T would be deep blue (uniformly zero error) in the colormaps, independent of both SNR and T_{true}; CNN_IMAGENET best approximates this ideal method when evaluated on the IMAGENET test dataset. See Figure 4 for further explanation and axis labeling.

medRxiv preprint doi: https://doi.org/10.1101/2023.01.11.23284194; this version posted January 12, 2023. The copyright holder for this preprint
(which was not certified by peer review) is the author/funder, who has granted medRxiv a license to display the preprint in perpetuity.
It is made available under a CC-BY 4.0 International license .

Dataset	Method	Bias	Precision	Error	SSIM
IMAGENET_TEST_1K	FIT_LOGLIN	0.041	0.108	0.418	0.145
	FIT_NLLS	0.065	0.143	0.386	0.159
	FIT_NLLS_BOUND	0.085	0.188	0.363	0.255
	FIT_NLLS_RICE	-0.177	0.272	0.545	0.148
	NN1D_IMAGENET	0.075	0.199	0.327	0.334
	NN1D_URAND	-0.009	0.162	0.321	0.333
	NN1D_SS_IMAGENET	0.100	0.218	0.396	0.166
	NN1D_SS_URAND	0.082	0.223	0.399	0.155
	NN1D_SS_INVIVO	0.050	0.191	0.425	0.215
	CNN_IMAGENET	-0.032	**0.039**	**0.102**	**0.669**
	CNN_URAND	-0.330	0.691	0.440	0.319
	CNN_SS_IMAGENET	0.114	0.228	0.396	0.185
	CNN_SS_URAND	0.084	0.225	0.391	0.175
	CNN_SS_INVIVO	**0.000**	0.130	0.250	0.413
URAND_TEST_1K	FIT_LOGLIN	0.071	0.100	0.480	0.158
	FIT_NLLS	0.079	0.130	0.457	0.149
	FIT_NLLS_BOUND	0.090	0.147	0.393	0.613
	FIT_NLLS_RICE	-0.203	**0.085**	0.621	0.359
	NN1D_IMAGENET	**-0.015**	0.142	**0.380**	**0.724**
	NN1D_URAND	-0.094	0.124	0.381	0.722
	NN1D_SS_IMAGENET	0.110	0.196	0.477	0.177
	NN1D_SS_URAND	0.094	0.197	0.479	0.118
	NN1D_SS_INVIVO	0.053	0.176	0.493	0.591
	CNN_IMAGENET	-0.118	0.086	0.445	0.630
	CNN_URAND	-0.510	0.191	0.586	0.467
	CNN_SS_IMAGENET	0.127	0.206	0.474	0.393
	CNN_SS_URAND	0.100	0.189	0.469	0.275
	CNN_SS_INVIVO	-0.301	0.097	0.479	0.571

Table S1 – Numerical values of the primary metrics plotted in Figure 4. The best values for each dataset (lowest bias, precision, and overall error; highest SSIM) are shown in **bold**.
